# Prediction of suitable brewing cuppages of Dahongpao tea based on chemical composition, liquor colour and sensory quality in different brewing

**DOI:** 10.1038/s41598-020-57623-5

**Published:** 2020-01-22

**Authors:** Sifeng Zhang, Yiqing Yang, Xiaofang Cheng, Kuberan Thangaraj, Emmanuel Arkorful, Xuan Chen, Xinghui Li

**Affiliations:** Institute of Tea Science, Nanjing Agricultural University, Weigang No.1, 210095 Nanjing, China

**Keywords:** Biochemistry, Plant sciences

## Abstract

Oolong tea is famous for its characteristic of durably brewing. To explore suitable brewing cuppages and the scientific methods to brew Oolong tea in multiple steeping process. Dahongpao tea (Zhengyan, Banyan and Zhouyan tea) is well known Oolong tea variety, brewed at 14 times and assessed its chemical composition, infusion colour and sensory quality in different brewing intervals. The results showed that Zhengyan tea (A3) had the best quality of steeping among the chosen tea. It could be brewed up to 10 cuppages with 80% sensory score. The chemical composition and tea infusion colour strength were higher in Zhengyan tea. Though, 70% caffeine leached within first three steeping. The Forest regression model revealed that the suitable brewing time ranges between 4 and 10 in the chosen Dahongpao tea variety. This study provides a scientific method and suitable steeping times for the drinking of different Dahongpao tea through dynamic analysis of quantity of chemical composition, infusion colour strength and sensory quality.

## Introduction

Oolong tea is well-known traditional Chinese tea. Dahongpao dated more than 350 years, is a representative of Oolong tea planted in Wuyishan, Fujian Province, China^1^. Oolong tea attracts both domestic and foreign consumers due to its quality and characteristic of durably brewing. Generally, Dahongpao tea are classified as Zhengyan (A1, A2, A3) Banyan (B1, B2, B3) and Zhouyan tea (C1, C2, C3) based on different growing environments. Zhengyan tea grows in Wuyishan Scenic Area along the Jiuqu river at high altitude, with lots of gravel in soil, which create good ventilation and moderate acidity of soil for its growing, resulting in excellent quality. Banyan tea is planted around Zhengyan tea region, on red soil with high acidity; moreover, the soil is mostly clay. Therefore, the quality of Banyan tea is not that good as Zhengyan tea. Zhouyan tea is distributed on hilly area with alluvial soil full of calcium, causing its quality not that good as Zhengyan and Banyan tea. Besides, the quality of tea infusion depends on the types and tea/water ration of tea, temperature of water, brewing duration and times^2–4^. To realize the best consumption quality experience of tea, scientific brewing methods are the key after cultivation and processing, especially for Chinese famous tea. Consumers are generally told that Oolong tea is suitable for continuous brewing up to more than 7 cuppages. This assertion is highly subjected to consumers’ discretion and has not been validated by any scientific research.

Sensory evaluation technique helps to determine the brewing cuppages and quality of tea infusion by its flavor strength. The flavor of tea infusion is determined by its chemical composition such as amino acids, caffeine, polyphenols and catechins. Several groups of compounds including phenolic compounds, purine alkaloids, amino acids, carbohydrates, nucleotides, organic acids and ions potentially contribute to the taste of tea. Sweetness of tea is mainly attributed to amino acids, as well as simple sugars such as sucrose, glucose and fructose; while bitterness, an important factor characterizing tea taste, is mainly due to catechins and caffeine. Catechins account for approximately 30% of the dry weight of tea leaves. Purine alkaloids, especially caffeine, are the other major contributors to the bitter taste of tea^5^. Caffeine induces bitter taste without activating bitter taste receptors. It also significantly enhances tea flavor. About 70% of the umami taste intensity of tea is contributed by amino acids, especially theanine and glutamic acid^6^.

Many researches focus on the effects of water temperature and steeping time on the quality of tea infusion^7^. The conclusions of these studies are usually obtained by analyzing the chemical content of tea infusion, or changes in sensory quality. Earlier studies reported that 2.5 g oolong tea brewed for 63 s with 120 mL of 98 °C water gives the best flavor^2,8^. Sua *et al*.^9^ also reported that oolong tea brewed at 95 °C for 3 min gives the best aroma and taste. However, these studies on brewing conditions, which only resulted from sensory analysis carried out through non-continuous brewing, cannot represent the essence of tea brewing. Thus, a dynamic analysis of the taste changes of tea infusion, taking into consideration the functional components of tea and their variations with brewing times, is needed. Therefore, the aim of this study was to explore the scientific methods and suitable brewing cuppages of Oolong tea by investigating the change rule of tea quality during brewing.

## Results

### Change of sensory quality and general acceptability of tea infusions during brewing

**Figure 1:**
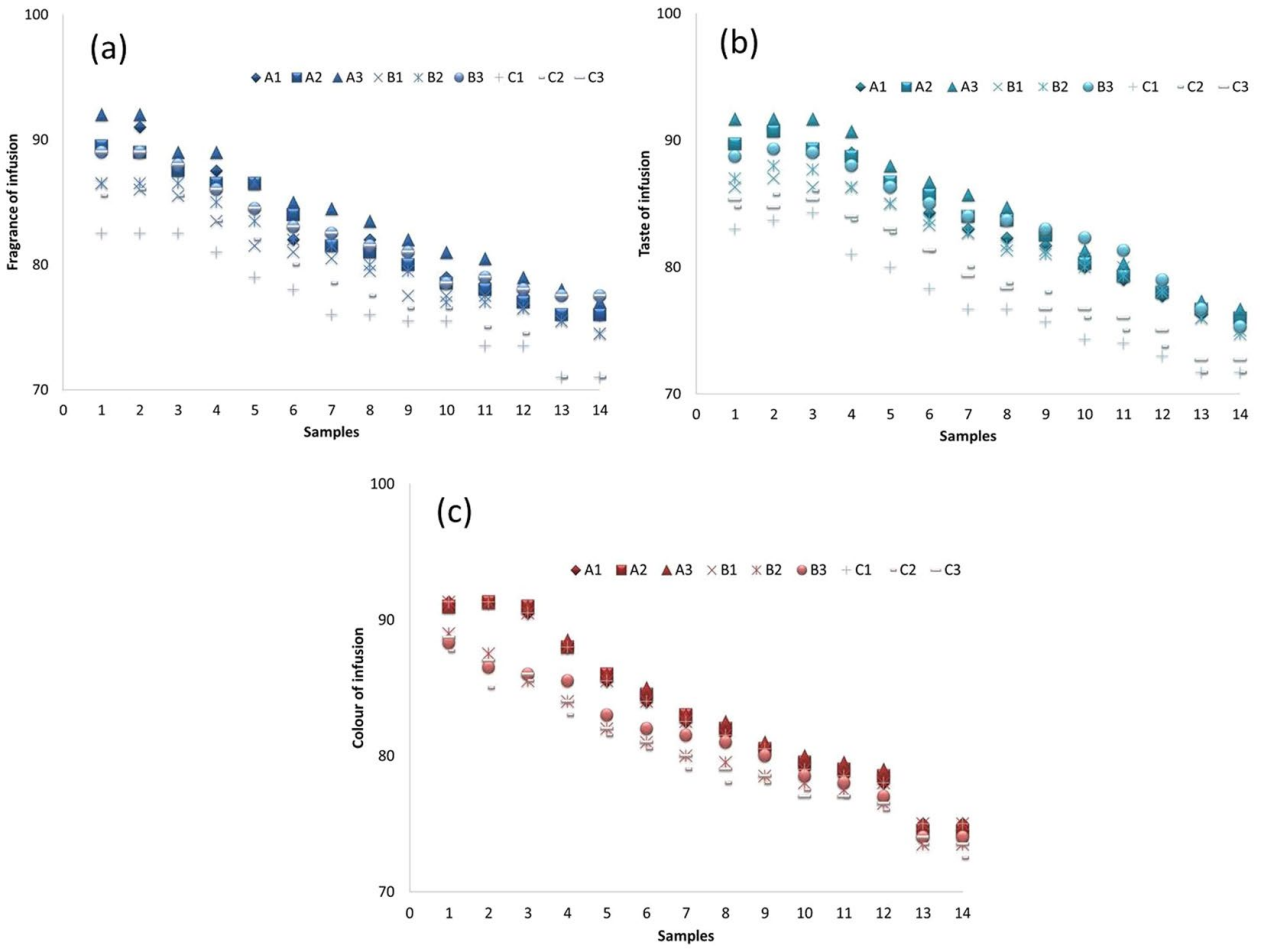
Sensory quality (Color, flavour and taste) of Zhengyan (A1, A2, A3), Banyan (B1, B2, B3) and Zhouyan (C1, C2, C3) tea infusion during the brewing process. The numbers 1–14 in figure represent the number of cuppages (brewing times). The numbers 0–100 represent the score of sensory evaluation. Data are presented as means ± SD.

**Figure 2:**
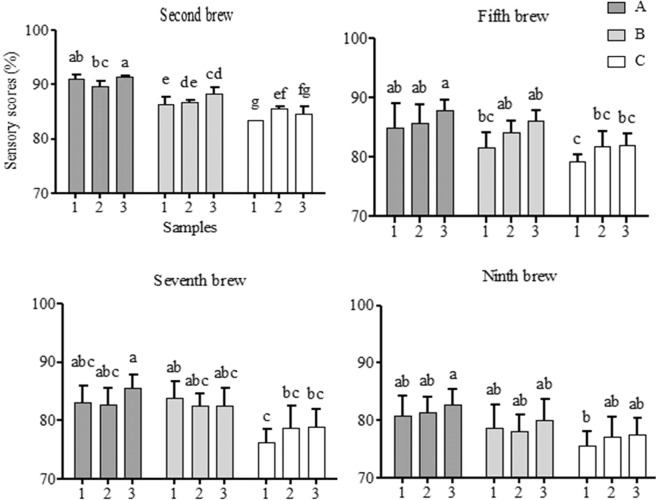
Overall acceptability of Zhengyan (A1, A2, A3), Banyan (B1, B2, B3) and Zhouyan (C1, C2, C3) tea samples. Data are presented as means ± SD. Different letters indicate significant differences at P < 0.05.

**Figure 3:**
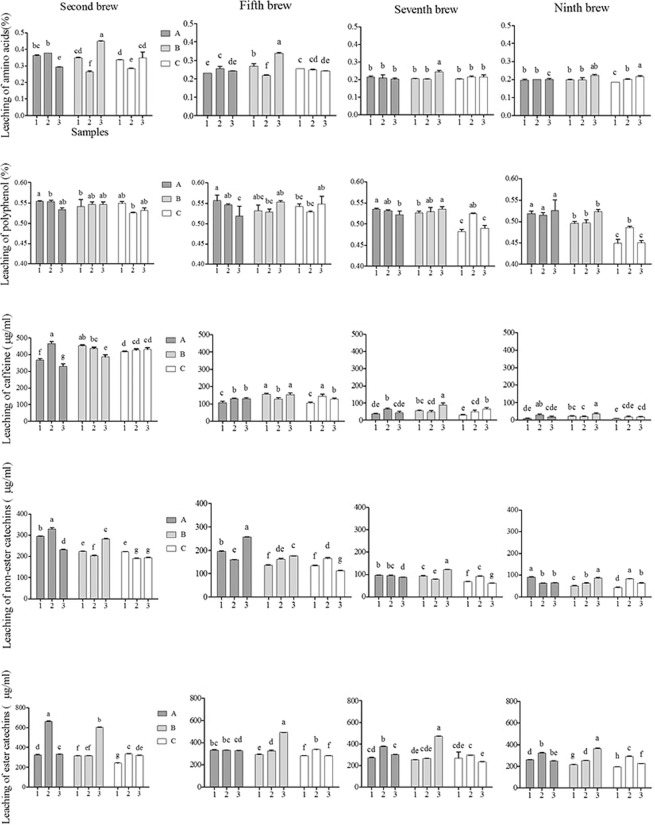
Leaching of amino acids, polyphenols, ester and non-ester catechins and caffeine contents of Zhengyan (A1, A2, A3), Banyan (B1, B2, B3) and Zhouyan (C1, C2, C3) tea samples at the second, fifth, seventh and ninth brew. Data are presented as means ± SD. Different letters indicate significant differences at P < 0.05.

Sensory quality (taste, aroma, and infusion colour) was presented in Fig. 1. Generally, taste, colour and aroma of infusion showed better quality during the first two brewing. During the whole brewing process, there is no significant difference among different brewing times and all samples kept sensory score above 70%. This indicated that the sensory quality of Dahongpao were stable during the whole brewing process, which ensured it could be brewed multiple times. Scores of sensory acceptability for the 2, 5, 7 and 9^th^ brew were presented in Fig. 2. The dynamic changes of sensory acceptability for all Dahongpao samples during the 14 times continuous steeping were showed in Table S1. In this study, all Dahongpao tea reached the highest sensory scores at the 2^nd^ brew, but when it came to the 9^th^ brew, only Zhengyan tea remained above 80%. While, Zhouyan tea sensory scores above 80% up to 5^th^ brew. There existed wide variation in general acceptability and suitability of brewing among Dahongpao samples, from different production areas. In addition, the overall acceptability ranged from 70 to 91% with the least and highest values observed in C3 and A3, respectively. This indicates that Dahongpao samples from different regions had different suitability of brewing. According to the standard of sensory evaluation, the sensory scores showed that A1, A2, A3, B1, B2, B3, C1, C2, C3 were suitable for brewing 9, 9, 10, 6, 7, 9 and 4, 6, 6 times respectively (Fig. 3). While the sensory scores for A3 and B3 could maintain above 80% for 10–11^th^ brew, respectively (Table S2). Further, C1 was suitable up to 4^th^ brew, while C2-C3 up to 6^th^ brew. These variations suggest the differences in brewing suitability that exist among tea samples in the same region and this might be due to different processing methods.

### Leaching rule of chemical composition in tea infusion during brewing

Leaching of total polyphenols, amino acids, caffeine, ester catechins, and non-ester catechins during 14 times brew in Dahongpao tea were determined and presented in Fig. S1. The leaching of chemical composition in tea infusion at the 2, 5, 7 and 9^th^ brew were showed in Fig. 3. Chemical composition increased at second brew, though the observation not significant (Tables S2–S6). The same trend was noticed in all the tea samples except C2 (Table S2), because leaching of ester catechins was highest at fourth brew. While the leaching of polyphenols of C2-C3 could only keep above 0.5% for about 6 times (Table S4). Combined with sensory evaluation, polyphenols in tea infusion was 0.5% below, the taste was bland. It indicated that the leaching of polyphenols was consistent with the trend of sensory quality. Leaching of amino acids in all the tea samples had no significant differences, all remained above 0.2% for 8^th^ brew around (Table S5). About 70% of caffeine leached during the 1–3^rd^ brew, and after 5^th^, there was almost no caffeine detected in the tea infusion. This might be due to it high solubility in water. Ester catechins and non-ester catechins from the 11–14^th^ brew-up and there is no significant differences were noticed in this study (Tables S2, S3). The results suggest that leaching of chemical composition in A1–A3 infusion remained at drinkable level for more times than B1–B3 and C1–C3. Furthermore, leaching of total chemical composition is not just dependent on location of Dahongpao tea and other factors also playing significant role.

### Change rule of tea infusion colour during multiple brewing

**Figure 4:**
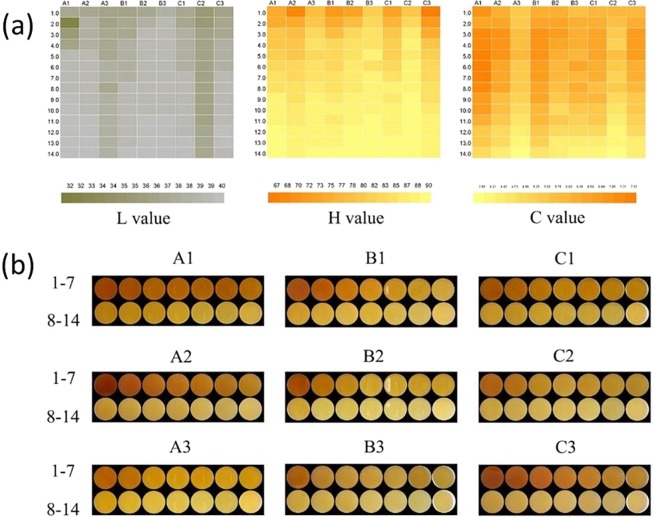
Heat map shows colour changes of tea samples during various brewing time. (**a**) L, H and C values represent colour changes during different brewing times of Zhengyan (A1, A2, A3), Banyan (B1, B2, B3) and Zhouyan (C1, C2, C3) tea samples. (**b**) Tea infusion colour changes during different brewing time (1–14 brewing times).

Colour variation of Dahongpao at different brewing times is presented as heat map in Fig. 4a. Colour difference values L, H and C were significantly correlated with the sensory quality and chemical composition of tea infusion. The brightness of tea infusion, represent by L value in the heat map, ranged from 0–100 indicating black to white. Generally, the L value gradually increased in the whole brewing process in all the tea samples. This implies that as brewing times increased, tea infusion became brighter and clear. All samples showed no significant differences in their brightness from 1–14^th^ brew. Though, A1 at 2^nd^ brew recorded the least value for L. H value in the heat map, revealed that tea infusion increased as brewing times increased, except for A3 at 5^th^ brew, B1 and B3 at 11 and 6^th^ brew, respectively, and C1 at 9^th^ brew, resulting in greener and less yellow colouration of tea infusion. It was observed that colour saturation of A1-A3 was higher than other samples, as shown by C value. Especially for Zhengyan tea A1, the C value maintained at more than 7^th^ brew. However, the C value of Banyan and Zhouyan tea infusion remained less than 7^th^ brew. Figure 4b shows the colour difference in all the tea samples during brewing. The results clearly revealed that A1 and B1 had the least change of infusion colour, while C2 recorded the highest, suggesting that A1 and B1 can resist colour change resulting from brewing better than other samples.

### Differential analysis and Principal Component Analysis (PCA) of tea infusion quality during multiple brewing

**Figure 5:**
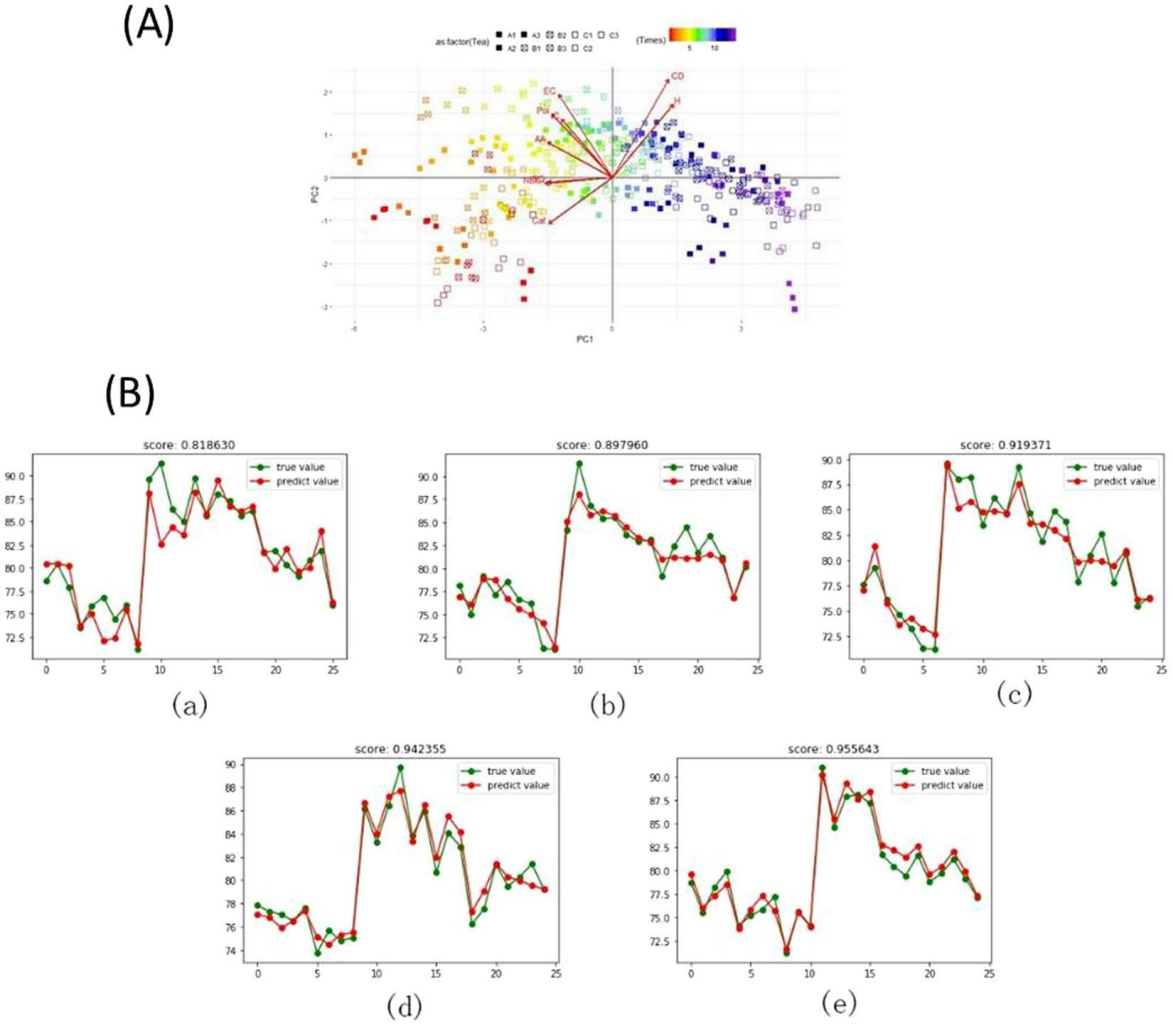
(**A**) Principal component analysis of Dahongpao tea samples. Changes in colour of icon represent change in brewing times. The separation between different samples represent significant difference between samples and brewing cuppages. Data are presented as means ± SD. (**B**) Forest random verification and P values of caffeine, catechins, total polyphenols, amino acids and sensory quality.

Further statistical analysis showed significant differences among Dahongpao tea infusion. Tea samples from different locations had varying levels of chemical composition and chemical leaching. Generally, chemical composition was higher in Zhengyan tea, followed by Banyan and Zhouyan tea infusion. Though, the chemical composition was varied within the samples of same groups (A1-A3, B1-B3 and C1-C3). PCA of tea infusion from different regions at different brewing times is shown in Fig. 5A. The results showed no clear cluster of tea samples and brewing times. Further differential analysis on sensory quality, chemical composition and infusion colour showed no clear clusters of tea samples at various brews (Figs. S2–S8). There was no clear separation of catechins (Figs. S2, S3). Total polyphenols in Zhouyan tea were partly separated from other regions from 7–14^th^ brew, but very low level (Fig. S4), while amino acid content was partly separated in Banyan and Zhengyan tea from 1–5^th^ and 13–14^th^ brew, respectively (Fig. S5). There was no separation in caffeine content throughout the entire brewing periods (Fig. S6), while colour change in Zhengyan was partly separated at lower levels from 2 to 14^th^ brew (Fig. S7). Separation of components for sensory quality followed similar trend as catechins (Fig. S8). The results suggest that distinction existed in sensory quality, tea colour and chemical composition during the whole brewing process, but no significant.

### Analysis of resistance of oolong tea to brewing

#### Construction of random forest model

Random Forest (RF) is an integrated statistical learning classification and regression algorithm that is easy to use and has good interpretability; in most cases, better results are obtained. Random forests are not sensitive to multicollinearity, thus its results for missing and unbalanced data are more solid^10^.

In this study, the factors affecting brewing times, the type of Dahongpao tea and the effects of sensory quality: caffeine, catechins, polyphenols and amino acids were used as features, and the sensory scores were fitted using the RF algorithm. Among them, the Dahongpao variety is used as a categorical variable, and it is one hot encoded, and other features are used as numerical variables. The specific modeling process is as follows:

1. First define the tea sensory score prediction training set ${x}_{i}\to {y}_{i}$, among which $x\leftarrow \{{I}_{i}^{1},{I}_{i}^{2}\cdots ,{I}_{i}^{n}\}$ is the feature vector of the tea sample, indicating the characteristics of the first sample.
2. Based on the determination of the training set, the construction of a single decision tree is as follows:
  1. Assuming that the number of training samples is *m*, the input samples for each decision tree are *m*, and the samples are randomly extracted from the training set.
  2. Assume that the number of training sample features is *n*, randomly select the number of *k* sample features of each decision tree, and then select the most appropriate feature from each feature to split. A regression tree corresponds to a partition of the input space (feature space) and an output value on the partitioning unit. Assuming that the input space has been divided into *k* units, *R*_1_, *R*_2_, *R*_*K*_, and there is a fixed output value in each unit of *R*_*m*_, the regression tree model can be expressed as:
    $$f(x)=\mathop{\sum }\limits_{{\rm{k}}=1}^{{\rm{K}}}\,{c}_{k}I(x\in {R}_{k})$$
    The squared error $\sum _{{x}_{i}\in {R}_{K}}\,({y}_{i}-f{({x}_{i})}^{2})$ can be used to represent the prediction error for the training data. It is easy to know that it is optimal when, *c*_*k*_ is the mean of all the actual values on the corresponding unit. The method of dividing the input space is to select the Jth variable *x*^(*j*)^and its value s as the segmentation variable and the segmentation point, and define two regions:
    $${R}_{1}(j,s)=\{x|{x}^{(j)}\le s\}$$
    $${R}_{2}(j,s)=\{x|{x}^{(j)} > s\}$$
    Then find the optimal segmentation j variable and the optimal segmentation point s, specifically, solve:
    $$\mathop{min}\limits_{j,s}[\mathop{min}\limits_{{c}_{1}}\sum _{{x}_{i}\in {R}_{1}(j,s)}{({y}_{i}-{c}_{i})}^{2}+\mathop{min}\limits_{c2}\sum _{{x}_{i}\in {R}_{2}(j,s)}{({y}_{i}-{c}_{2})}^{2}]$$
    Traverse the variable j, scan the segmentation point s for the fixed segmentation variable j, and select the pair (j, s) that makes the above formula reach the minimum value. With the selected pair (j, s).
  3. Continue to divide the two sub-regions obtained by the above split until the number of samples in the node is less than the threshold or no more features, and no pruning is needed during the splitting process.
3. The generated multiple decision trees are composed of random forests, and the regression results obtained by all decision trees are taken as the final model output.

In order to make full use of the data, a 50% cross-validation was used for the random forest regression model. Evaluation index selection goodness of fit of *R*^2^ to test the degree to which the regression model fits the sample data. The goodness of fit is between 0 and 1. The higher the goodness of fit, the higher the degree of interpretability of the model. The formula for calculating the goodness of fit is as follows:

$${R}^{2}=\frac{{\sum }_{i=1}^{n}{({\hat{y}}_{i}-\bar{y})}^{2}}{{\sum }_{i=1}^{n}{({y}_{i}-\bar{y})}^{2}}$$

Where: *y*_*i*_ is the actual observation, that is, the actual sensory evaluation index; ${\hat{y}}_{i}$ the predictive sensory evaluation index for the model. $\bar{y}$ is the actual sensory evaluation of the mean of the index.

### Random forest model on brewing times

A random forest consists of multiple decision trees, and each node in the decision tree is a division of the feature space. Nodes (optimal conditions) can be determined using average impurity. For classification problems, Gini impurity or information gain is usually used. For regression problems, variance or least squares fit is usually used. In this study, the features were ranked according to the average impurity, and the results were as

$$f(x)=\mathop{\sum }\limits_{{\rm{k}}=1}^{{\rm{K}}}\,{c}_{k}I(x\in {R}_{k})$$

Where: *y*_*i*_ is the actual observation, that is, the actual sensory evaluation index; ${\hat{y}}_{i}$ the predictive sensory evaluation index for the model. $\bar{y}$ is the actual sensory evaluation of the mean of the index.

The validation showed that sensory quality evaluation was the most accurate predictor of change associated with brewing times, followed by amino acid, total polyphenol, catechins, while caffeine was the least indicator (Fig. 5B). The results also revealed that the fit of the eigenvalues for all components under study was greater than 0.8. It was predicted from this result, in combination with analyses of leaching of chemical composition and sensory quality, that suitable brewing cuppages for Dahongpao tea A1, A2, A3, B1, B2, B3, C1, C2 and C3 was 9, 9, 10, 7, 7, 9, 4, 6, 6, respectively. The obtained results suggests that A1, A2 and A3 are more suitable for drinking followed by B1, B2, B3 and C1, C2, C3.

## Discussion

Oolong tea is generally considered to brew up to 14 cuppages. In order to scientifically verify this assertion, the sensory quality, contents of polyphenols, amino acids, caffeine, and catechins in Dahongpao samples, one of typical Oolong tea, were determined. In addition, the leaching rule of these chemical composition was assessed to explore the suitability during multiple brewing times of Dahongpao.

Flavor, aroma and appearance are the basic sensory components of tea^11^. The present study evaluated selected attributes of tea in order to understand the sensory profile of Dahongpao tea. In the present study, the overall acceptability score was between 70 and 91%, and was parallel with colour, taste and aroma of tea infusion. This indicates that Dahongpao has characteristic of resistance to lasting steeping. Saklar *et al*.^12^ reported a positive relationship between overall acceptability and sensory quality; however, in their report, general acceptability ranged from 10 to 85% with 85 °C water. This report differs from the present study, it might be the variations in brewing temperatures, timing, and also to the fact that the tea samples employed in both studies have different levels of chemical compositions. In the present study, sensory quality of all tea samples ranged between 71–92%. In contrast, Saklar *et al*.^12^ reported a sensory quality scores between 1–87%. The disparity in these results could be attributed to the fact that the temperature of water and method of brewing used in both experiments differ. While, 85 °C water for up to 45 min of brewing, the present study employed 100 °C water for up to 38 min in multiple way brewing.

In this study, sensory quality gradually decreased from the 4^th^ brew as brewing times increased. This observation is consistent with report of Sua *et al*.^9^ and observed that sweetness, aroma and general sensory quality of tea decreased when soaking time extended from 3 to 10 min at 100 °C brewing. Further, brewing time reached 30 and 45 min, sensory scores for tea infusion were very low^12^. This could be due to its perceived bitter taste. The astringency can be attributed to gallated tea flavonols and theaflavins, particularly mono- and di-gallate, catechins, caffeine and some amino acids (such as arginine and alanine) that contribute to the bitterness of green infusions^13^. Since tea aroma, a major component of sensory quality, is dependent on volatiles, long brewing period may have resulted in the loss of some volatiles in tea solution^14^. Therefore, it can be speculated that decomposition of aroma compounds occurs during brewing at higher temperatures. In this study, longer brewing period (38 min) at higher temperature (100 °C) had a negative influence on sensory quality of tea infusion. Continuous brewing of tea beyond 15 min still undergoes chemical leaching, yet results in decrease in sensory quality; thus, not suitable for continuous drinking^15^. This report contradicts with result of in this study and the discrepancy could be attributed to different brewing process.

Catechins is the main component of polyphenols^11^. In the present study, ester catechins leaching decreased as brewing times increased, and reached 95 µg/mL at the fourteenth brew. Increasing brewing time beyond 20 min in a continuous brewing process was not effective in extracting more catechins into hot water during a 45 min continuous brewing period^12^. They also reported that at 75 °C, the total catechins content increased with increasing brewing time. In contrast, the present study brewing time beyond 30 min in multiple brewing process resulted in leaching 95 µg/mL catechins. However, several studies have reported that as brewing time increased, chemical composition including catechins decrease, and this closely associated with our findings. This could be due to epimerization of the chemical composition in tea over long period of brewing time. Other studies have reported that catechins leaching in green tea^12^ and white tea^16^ reaches its limit after 10–15 min of continuous brewing. Contrary to the earlier reports, the present study revealed that catechins leaching in Dahongpao tea infusion was still detectable at 38 min of brewing. This indicates that catechins leaching in Dahongpao tea infusion is steady and lasting.

Pérez-Burillo *et al*.^8^ observed that at temperatures higher than 80 °C, caffeine increased almost exponentially when infusion time was above 10 min. This suggests that temperature and infusion time are the main determining factor in extracting chemical composition^3,12,17^, and could explain the discrepancies in the results in the present and previous studies. This could also be attributed to oxidation reaction during longer extraction times of caffeine. Furthermore, in this study, the amount of caffeine leaching was still detectable until 10^th^ brew, and this is contrary to other reports^12,16^. This could reveal the reason why Dahongpao tea infusion could be brewed up for many times with good tasting. Shao *et al*.^18^ found that the total amount of polyphenols and free amino acids leaching in West Lake Longjing 43 tea infusion increased with increasing brewing times. This was attributed to the fact that West Lake Longjing 43 is a green tea with high raw material tenderness and has a fast leaching rate of chemical components; thus, not suitable for multiple brewing. However, in the present study, leaching of chemical composition was higher in the first and second steeps and rapidly decreased as brewing times increased.

The lightness (L), chroma (C) and hue angle (H) values of the tea infusions at different brewing stages displayed noticeable variation. In the present study, the L value generally increased as brewing times increased. This implies that the L values of tea infusions are significantly influenced by duration and times of brewing. The chroma, that is, the intensity or saturation, is considered as the degree of colour relative to a similarly illuminated neutral grey. It was also observed that colour saturation, as shown by C value, gradually decreased resulting in lower saturation as brewing times increased. Moreover, the hue of tea infusion increased as brewing times increased. These colour changes may be due to oxidation of catechins in the infusions during brewing^19^.

The infusion colour of oolong tea is generally reddish-brown in moderate to heavy fermented oolong and dark greenish colour in light fermented oolong^20^. Colour-determining compounds in light-fermented tea are composed of flavonols, flavones and oxidized polyphenolic compounds such as theaflavins (TFs) and thearubigins (TRs), while the major colour-determining compounds in moderate and heavy-fermented oolong tea are TRs and their oxidized polymers^21^. These could account for the discrepancy in infusion colour as brewing times increased, as observed in this study. In addition, homobisflavin compounds such as homobisflavin A, B, theasinensin D, E, F, G and thenin are related to the colour of oolong tea infusion^20^. Colour change is the most intuitive way to judge the quality of tea infusion. In this study, tea infusion colour changed from orange red or orange to light yellow. During the fermentation process, the green colouration of tea leaves changes to coppery brown^21^. According to the Dahongpao testing standard, Dahongpao tea infusion with orange or orange red colour is suitable for drinking. Theaflavins are responsible for the brightness, briskness and quality of tea infusion; while the colour, taste and body are determined by the content of TR. In addition, TF and TR are responsible for orange-red and reddish-brown colour pigments, respectively^22,23^. Coloured TFs and TRs are produced by the enzymatic oxidation and condensation of catechins in green leaf during fermentation process^19^. The ratio of TFs and TRs determines the gloominess of black tea. This is produced by the decomposed products of chlorophyll, protein, pectin, sugar and phenolic compounds which form during the brewing process and accumulate on the surface of tea infusion^20^.

Tea infusion colour is mainly determined by the chlorophyll content and the ratio of chlorophyll A, responsible for dark green colouration, to chlorophyll B, responsible for yellowish-green colouration^24^. The degradative products of chlorophyll (pheophytin and pheophorbide) may be responsible for the dark colouration of tea infusion. The degradation is activated by the chlorophyllase enzyme, high temperature and high humidity^20^. The long period of exposure of tea samples through multiple brewing method in the present study might have contributed to the change in infusion colour in the present study. Besides the water-soluble anthocyanins, the yellow colouration in green tea infusion is mainly determined by the water soluble flavonols, including kaempferol, quercetine, isoquercetin, myricetin, myricitrin, rutin, kaempferitrin, and flavones including apigenin, isovitexin, vitexin, saponarin, vicenin-2, as well as their glycosides^20^. These chemical compounds were released from the tea leaves into the infusion during the brewing process at high temperature, and this could contribute to the yellow colouration of tea infusion as brewing time increased, as observed in this study.

## Methods

### Reagents, standard chemicals and solvents

Chromatographic grade reagents, standards and solvents were used in this study. These included gallic acid and L-amino acid standards (Shanghai Ruiyong Biotech Co., Ltd.); Caffeine, gallocatechin (GC), epicatechin gallate (ECG), epicatechin (EC), gallate (EGCG), epigallocatechin (EGC), catechin gallate (CG), gallocatechin gallate (GCG), catechin (C), epigallocatechin standards and Folin-Phenol (Shanghai Yuanye Biotechnology Ltd.); Ninhydrin (Sinopharm Group Chemical Reagent Co., Ltd.); pure water (Hangzhou Wahaha Co., Ltd. Zhejiang, China).

### Tea samples

**Figure 6:**
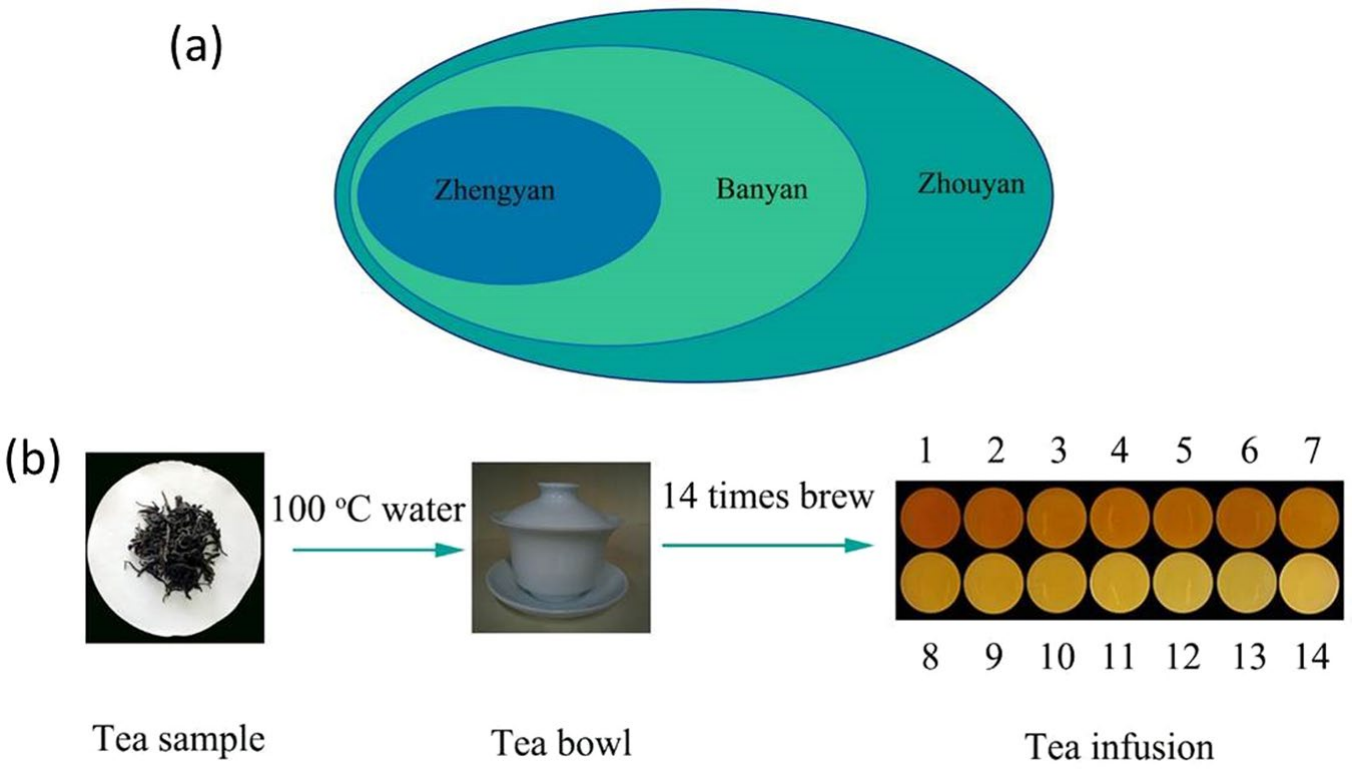
(**a**) Dahongpao tea growing area divisional map. Zhengyan (A1, A2, A3), Banyan (B1, B2, B3) and Zhouyan (C1, C2, C3) tea are distributed like the circle picture. (**b**) Diagrammatic presentation of tea brewing method. 5 g of each tea sample was boiled in 100 °C pure water for 14 times.

Nine Oolong tea samples made by *Camellia sinensis* cv. planted in Wuyishan, Fujian Province (China) were collected between March-April, 2017. Zhengyan tea (A1, A2 and A3), Banyan tea (B1, B2 and B3), and Zhouyan tea (C1, C2 and C3) were procured from three different regions and same were used for further experimental purpose (Fig. 6a). All tea samples were stored in vacuum until use.

### Preparation of tea infusions

According to the local standard of Wuyi rock tea sensory evaluation, 14 cuppages (Fig. 6b) were prepared from 5 g tea sample with 110 mL of 100 °C pure water, with total steeping duration of 38 min (Table 1). Sensory evaluation and the detection of chemical composition were conducted on the tea infusion.

**Table 1:** The time setting of multiple brewing.

BT	1	2	3	4	5	6	7	8	9	10	11	12	13	14
T/s	20	30	45	60	80	100	120	150	180	220	260	300	340	380

BT represents steeping times; T/s represents time in seconds.

### Detection of catechins and caffeine

According to the national standards^25^, catechins and caffeine were detected by high performance liquid chromatography (Shimadzu Co. Ltd., Japan) using C18 column at 35 °C. Mobile phase A with 90 mL acetonitrile, 20 mL acetic acid and 2 mL EDTA; mobile phase B with 800 mL acetonitrile, 20 mL acetic acid and 2 mL EDTA were employed. Tea infusions were filtered through a 0.22 µm filter before injection. The tea chromatographic conditions were as follows: mobile phase flow rate of 1 mL/min, parameter setting of 0.01 min for 100% A; 10 min 100% A; 10.01 min 100% A, 25 min 68% A, 32% B; 25.01 min 68% A, 32% B; 35 min 68% A, 32% B; 35.01 min 100% A and 45 min 100% A. A blank run was conducted upon stabilization of flow rate and column temperature. A 10 µL mixed standard series working solution was pipetted into the HPLC for detection and used as standards. After standardization, 10 µL sample infusion was used for the detection. The test solution was quantified with appropriate standards.

### Colour measurement of tea infusions

Tea infusion colour was measured using 3nh colorimeter (Shenzhen Sanenshi Technology Co., Ltd.). HemI and CIE L*a*b * were used to distinguish the tea infusions described by Sui *et al*.^26^ Briefly, the coordinates of L*C*H° were calculated from L*a*b* values and calculated based on the previously described^26^. L* represents the lightness measuring brightness with 100 and 0 equaling to absolute white and absolute black, respectively. Chroma (C*) measures intensity or saturation. H° indicates hue angle which is expressed on a 360° grid, with 0° and 180° corresponding to + a* axis (red) and a* (green), respectively, and 90 and 270 for the + b* axis (yellow) and b* (blue), respectively. Analysis of the lightness (L*), chroma (C*) and hue angle (H°) values of each infusion was done to measure the colour changes at all brewing times.

### Detection of amino acids and total polyphenols

Free amino acids were detected by ninhydrin colorimetry and read with spectrophotometer at 570 nm using L-theanine as standard curve, as described by Tu *et al*.^27^ Total polyphenol content was detected by the Folin-Ciocalteu method as previously described^28^, and read with ultraviolet spectrophotometer (Yuan Xi Instrument Co., Ltd., Shanghai) at 765 nm.

### Sensory evaluation of dahongpao tea infusions

Sensory evaluation procedure was conducted in accordance with the Chinese National Standard as GB/T 23776-2009 Methodology of Sensory Evaluation of Tea. Fifteen panelists with experience in tea sensory evaluation were participated in this study. Before the evaluation, 30 min training session was conducted to familiarize them with the sensory attributes of Dahongpao tea. Tea infusions at 14 brewing cuppages were then served in evaluation cups respectively, and presented to each of the panelists. Scores was given for colour, aroma and taste on a percentile system with 0 = extremely dislike and 100 = extremely like. Participants rinsed their mouth with pure water before the next evaluation^29^.

Each evaluation factor adopted a percentage system, taste 40%, aroma 40%, colour 20% and the total was computed for overall acceptability. The evaluation for each tea sample was re-verified three times, and the average taken as final score. The standard of sensory evaluation of Dahongpao tea was shown in Table 2.

**Table 2:** The standard of sensory evaluation of Dahongpao tea.

Sensory factors	Description of sensory characteristics	Corresponding scores (%)
aroma	strong fruity flowery, elegant fragrant, long-lasting,	90–100
	fruity flowery, long-lasting	80–90
	short fragrant	70–80
	unobvious short aroma,	60–70
	weak aroma	50–60
	no aroma	40–50
taste	thick and mellow, Lubricating, obvious sweet after taste	90–100
	mellow and thick, sweet after taste	80–90
	mellow and thin, sweet after taste	70–80
	mellow and thin, unobvious sweet after taste	60–70
	plain and thin	50–60
	dull like water	40–50
color	orange yellow or orange red, bright	90–100
	orange yellow, light bright	80–90
	yellow, light bright	70–80
	yellow	60–70
	yellowish	50–60
	light	40–50

### Data analysis

One-way analysis of variance (ANOVA) with Duncan test was used to obtain significant variations (P < 0.05). The relationship between chemical components and evaluation results were performed by computing Pearson correlation coefficient at P < 0.05 confidence level. Obtained data were subjected to principal component analysis (PCA). The results of suitable brewing cuppages were obtained by The Random Forest model. All experiments were repeated three times and results presented as means ± standard deviation (SD).

### Ethics statement

The protocol used in the present study was approved by the Nanjing agricultural university ethical committee (SYXK (Jiangsu)-2017-0007) and all experiments were performed in accordance with the approved guidelines. Further, verbal informed consent was obtained from every participant in the study. No local regulations or laws were overlooked throughout the course of the investigation.

## Supplementary information

Supplementary Information Doc.-Table.

Supplementary Information Doc.-Figure.

## Data Availability

All data generated or analyzed during this study are included in this published article (and its Supplementary Information files).
