# Supplementary material for: Prediction of suitable brewing cuppages of Dahongpao tea based on chemical composition, liquor colour and sensory quality in different brewing
Source: Sci Rep. 2020 Jan 22;10:945. doi: 10.1038/s41598-020-57623-5 (PMC6976566; doi:10.1038/s41598-020-57623-5)
Supplement: Supplementary file 1 — Supplementary Information Doc.-Table. [file 41598_2020_57623_MOESM1_ESM.docx]

| **Prediction of suitable brewing cuppages of Dahongpao tea based on chemical composition, liquor colour and sensory quality in different brewing**    Sifeng Zhang, Yiqing Yang, Xiaofang Cheng, Kuberan Thangaraj, Emmanuel Arkorful, Xuan Chen, Xinghui Li*  Institute of Tea Science, Nanjing Agricultural University, Weigang No.1, 210095, Nanjing, China  * Corresponding author: Xinghui Li  Email: lxh@njau.edu.cn  Tel/fax: +86 25 8439 6651  Sifeng Zhang 2016104090@njau.edu.cn  Yiqing Yang 14216111@njau.edu.cn  Xiaofang Cheng 14217131@njau.edu.cn  Kuberan Thangaraj tku2010@gmail.com  Emmanuel Arkorful emmamidnite@hotmail.com  Xuan Chen chenxuan@njau.edu.cn  Xinghui Li lxh@njau.edu.cn  **Table S1.** Scores of general sensory quality (%) | | | | | | | | | | |
| --- | --- | --- | --- | --- | --- | --- | --- | --- | --- | --- |
| BT/S | A1 | A2 | A3 | B1 | B2 | B3 | C1 | C2 | C3 |  |
| 1 | 89.60±0.5ab | 89.35±1.75ab | 91.35±0.75a | 86.45±0.25a | 86.15±0.45ab | 88.00±0.10a | 83.25±0.25ab | 85.00±1.00a | 85.25±0.75a |  |
| 2 | 90.95±0.95a | 89.70±1a | 91.40±0.20a | 86.4±1.30a | 86.85±0.25a | 88.25±1.25a | 83.50±0.00a | 85.65±0.25a | 84.65±1.25b |  |
| 3 | 88.05±2.35abc | 87.85±2.15ab | 89.70±2.40ab | 85.4±2.30ab | 86.15±1.15ab | 87.30±2.80ab | 83.80±1.10a | 85.50±0.40a | 84.80±0.20c |  |
| 4 | 88.05±2.45abc | 87.15±2.45abc | 89.20±2.00ab | 83.7±2.60abc | 84.65±1.52abc | 85.95±2.85abc | 80.70±0.70bc | 82.95±1.95ab | 83.25±1.25ab |  |
| 5 | 84.85±4.15bcd | 85.70±3.2abcd | 86.20±1.76abc | 81.65±2.55abcd | 83.10±1.90abcd | 84.10±1.73abcd | 79.15±1.15cd | 81.70±2.70abc | 81.95±1.95abc |  |
| 6 | 82.45±3.75cde | 83.80±4.1bcde | 84.50±2.14bcd | 80.5±2.89abcde | 81.90±2.08abcde | 82.90±2.22abcd | 77.90±1.90de | 80.30±3.30bcd | 80.50±2.50bcd |  |
| 7 | 81.75±4.65def | 81.65±4.35cdef | 83.60±2.29bcde | 79.45±2.90bcde | 80.85±2.05bcde | 81.90±2.96abcde | 76.25±2.25ef | 78.80±3.80bcde | 79.00±3.00cde |  |
| 8 | 81.20±4.2def | 81.35±4.15def | 82.65±2.32cdef | 79.5±2.21bcde | 79.70±2.14cdef | 81.20±2.14bcde | 75.90±2.50efg | 77.80±3.80cdef | 78.05±3.05cde |  |
| 9 | 80.20±4.3def | 80.30±4.2def | 81.40±2.67cedf | 79.25±2.76bcde | 79.15±2.14cdef | 80.65±2.59cde | 75.50±2.50efg | 77.15±3.55cdef | 77.40±2.90de |  |
| 10 | 78.75±3.85ef | 78.60±3.5ef | 80.40±2.67cdef | 78.15±2.12cde | 77.60±1.97def | 79.25±2.50de | 75.00±2.00fg | 76.10±3.10cde | 77.00±3.10def |  |
| 11 | 78.25±3.35ef | 77.85±2.75f | 79.90±2.22cdef | 77.85±1.91cde | 77.30±1.74ef | 79.15±2.44de | 74.10±1.50fg | 75.20±2.60efg | 76.80±3.20def |  |
| 12 | 77.05±3.15ef | 77.15±30.5f | 78.50±1.90def | 76.65±3.23de | 76.50±2.18ef | 77.65±2.01de | 73.25±1.25gh | 73.75±1.75fgh | 76.00±3.00efg |  |
| 13 | 75.65±2.15f | 75.85±1.85f | 77.20±1.67ef | 74.80±0.99e | 75.05±1.026ef | 76.15±1.58e | 71.25±0.75h | 71.25±0.75gh | 73.25±0.25fg |  |
| 14 | 75.65±2.15f | 75.85±1.85f | 76.80±1.56f | 74.45±1.45e | 74.15±0.71f | 75.75±1.52e | 71.17±1.04h | 70.50±0.50h | 72.50±0.50g |  |

General sensory acceptability of Zhengyan (A1, A2, A3), Banyan (B1, B2, B3) and Zhouyan (C1, C2, C3) tea samples. BT represents steeping times (cuppages); S represents Samples. Data are presented as means ± SD of three replicates. Different letters indicate significant differences at P < 0.05.

| **Table S2.** Leaching of ester catechins (μg/mL) | | | | | | | | | | |
| --- | --- | --- | --- | --- | --- | --- | --- | --- | --- | --- |
| BT/S | A1 | A2 | A3 | B1 | B2 | B3 | C1 | C2 | C3 |  |
| 1 | 286.37±3.75e | 541.57±3.61b | 166.77±1.70k | 264.07±3.79d | 302.67±3.06d | 405.20±2.97g | 198.57±2.32de | 291.20±3.70e | 287.20±1.97b |  |
| 2 | 325.40±5.23c | 661.70±3.75a | 332.20±3.54b | 314.17±3.67b | 317.60±1.82c | 603.03±3.33g | 240.67±3.06bc | 296.23±2.14d | 320.17±1.26a |  |
| 3 | 304.90±2.62d | 489.97±2.86c | 355.73+2.11a | 322.40±5.81a | 323.30±2.07b | 542.57±2.70b | 234.10±3.35 c | 336.67±2.08b | 302.87±3.01ab |  |
| 4 | 369.13±4.17a | 472.33±4.93d | 330.17±0.72b | 293.50±3.90c | 345.67±2.08a | 523.50±3.12c | 224.30±4.00cd | 384.87±3.84a | 293.87±2.20ab |  |
| 5 | 332.97±5.45b | 412.67±2.52e | 327.83±2.57b | 264.17±3.33d | 326.00±6.00b | 491.33±2.31d | 282.93±2.67a | 338.60±1.97b | 282.83±3.47b |  |
| 6 | 284.20±5.57e | 376.83±3.40f | 303.43±3.57c | 253.53±2.45e | 274.27±4.08e | 487.33±5.03d | 243.30±3.46bc | 331.73±2.68c | 221.53±1.67d |  |
| 7 | 274.70±4.42f | 374.10±3.05f | 301.50±1.50c | 244.23±2.93f | 267.07±2.76f | 471.33±1.53e | 268.10±58.55ab | 296.73±1.86d | 233.90±1.014e |  |
| 8 | 264.33±3.75g | 333.42±2.65g | 276.10±4.87d | 228.53±3.07g | 265.50±2.18f | 414.33±4.51f | 226.67±2.47c | 267.33±3.05f | 225.87±2.80f |  |
| 9 | 260.10±5.31g | 324.60±2.42h | 248.73±3.25e | 214.27±3.10h | 255.50±3.04g | 364.67±3.51h | 195.67±1.15e | 292.33±2.52de | 225.90±0.85h |  |
| 10 | 230.80±1.49h | 295.73±1.72i | 231.33±3.69f | 206.17±3.75i | 223.67±4.04h | 327.83±2.25i | 181.90±1.15ef | 247.67±2.52g | 182.67±2.52i |  |
| 11 | 228.10±3.25i | 268.83±3.75j | 220.33±4.16g | 176.37±3.00j | 200.00±2.00i | 314.77±2.40j | 163.10±2.69fg | 235.33±2.52h | 152.57±2.50j |  |
| 12 | 190.10±1.85j | 231.20±2.25k | 205.10±3.60h | 170.70±1.21k | 197.27±3.10i | 283.17±2.36k | 155.50±4.27fg | 214.80±3.50i | 141.73±3.04k |  |
| 13 | 184.07±4.38ij | 222.60±3.37i | 199.80±1.51i | 170.03±2.83k | 184.30±2.23j | 257.83±5.35i | 141.40±4.08gh | 220.27±1.91j | 142.33±5.69i |  |
| 14 | 178.57±3.27j | 200.43±2.40m | 190.33±3.51j | 148.27±2.34i | 171.67±3.51k | 242.17±5.11m | 123.33±3.13h | 200.37±2.51k | 94.67±3.97f |  |

Leaching of ester catechins of Zhengyan (A1, A2, A3), Banyan (B1, B2, B3) and Zhouyan (C1, C2, C3) tea samples. BT represents steeping times (cuppages); S represents Samples. Data are presented as means ± SD of three replicates. Different letters indicate significant differences at P < 0.05.

| **Table S3.** Leaching of non-ester catechins (μg /mL) | | | | | | | | | | |
| --- | --- | --- | --- | --- | --- | --- | --- | --- | --- | --- |
| BT/S | A1 | A2 | A3 | B1 | B2 | B3 | C1 | C2 | C3 |  |
| 1 | 303.17±3.25a | 255.33±3.06b | 118.80±1.59e | 166.67±3.21c | 204.33±4.04a | 192.17±2.02c | 167.87±1.80b | 176.67±1.53c | 183.43±1.50b |  |
| 2 | 294.33±3.06b | 329.33±6.03a | 232.33±2.52b | 223.00±2.65a | 202.9±2.59a | 282.80±2.55a | 221.87±1.63a | 191.27±1.10b | 194.13±2.01a |  |
| 3 | 234.67±3.51d | 243.33±4.16c | 154.23±3.91d | 190.20±2.03b | 182.57±2.5b | 212.57±2.50b | 162.5±2.50c | 158.33±2.52e | 141.77±1.67c |  |
| 4 | 273.00±2.65c | 164.00±4.00d | 212.63±2.12c | 121.63±1.52d | 131.57±1.91d | 175.33±2.52e | 163.53±1.86c | 212.33±3.21a | 128.67±0.58d |  |
| 5 | 194.67±2.31e | 158.00±2.00e | 256.33±1.53a | 135.00±3.00e | 161.00±3.61c | 175.47±0.50d | 133.00±2.65d | 163.67±4.04d | 111.40±2.25e |  |
| 6 | 117.33±4.73f | 108.67±0.58f | 96.17±2.02f | 72.00±2.65d | 92.33±2.52e | 142.27±2.53e | 76.60±2.00e | 142.93±2.61f | 72.87±3.01f |  |
| 7 | 97.00±10.00g | 95.00±3.00g | 88.10±1.15g | 92.60±2.95g | 77.93±2.61f | 121.83±1.76f | 67.67±1.52f | 91.90±1.15g | 60.17±2.02g |  |
| 8 | 83.33±3.51i | 80.00±2.00hj | 71.53±1.50h | 55.03±0.84f | 72.40±1.77g | 104.27±3.74g | 59.67±1.53g | 82.90±1.82h | 55.17±3.01h |  |
| 9 | 91.33±1.15h | 63.27±1.90i | 64.33±1.53i | 51.17±1.61h | 62.87±2.80h | 87.00±2.00h | 43.20±2.88i | 92.90±1.82g | 62.9±2.59g |  |
| 10 | 62.67±2.08j | 72.27±2.41j | 57.10±1.40j | 49.00±1.00h | 70.90±1.01g | 84.00±2.65h | 47.67±1.53h | 72.00±2.00i | 37.27±2.25i |  |
| 11 | 52.47±1.36k | 47.53±2.23i | 54.23±3.36j | 26.33±1.53i | 46.80±1.59j | 57.57±1.40i | 28.50±3.04j | 56.33±1.53j | 26.00±2.00j |  |
| 12 | 37.10±2.29i | 36.00±1.00i | 42.67±2.57k | 24.50±1.80j | 41.37±1.59j | 46.60±1.77j | 26.70±1.25j | 45.93±1.79k | 21.17±1.04j |  |
| 13 | 31.47±1.75m | 31.5±1.80m | 41.07±3.38k | 20.17±1.89k | 33.23±2.80k | 25.93±1.90k | 21.27±1.10k | 40.77±1.08i | 20.10±1.45k |  |
| 14 | 28.13±1.40m | 25.27±0.00m | 32.60±1.83i | 17.00±1.00i | 28.40±1.83i | 10.90±1.01i | 18.33±1.55k | 36.33±1.53m | 17.43±1.69k |  |

Leaching of non-ester catechins of Zhengyan (A1, A2, A3), Banyan (B1, B2, B3) and Zhouyan (C1, C2, C3) tea samples. BT represents steeping times (cuppages); S represents Samples. Data are presented as means ± SD of three replicates. Different letters indicate significant differences at P < 0.05.

| **Table S4.** Leaching of polyphenols (μg /mL) | | | | | | | | | | |
| --- | --- | --- | --- | --- | --- | --- | --- | --- | --- | --- |
| BT/S | A1 | A2 | A3 | B1 | B2 | B3 | C1 | C2 | C3 |  |
| 1 | 0.55±0.005a | 0.55±0.001a | 0.52±0.01ab | 0.52±0.003bc | 0.52±0.029cd | 0.53±0.002cd | 0.53±0.003bc | 0.52±0.003ab | 0.53±0.006a |  |
| 2 | 0.55±0.002a | 0.55±0.003a | 0.53±0.005a | 0.53±0.035a | 0.55±0.006a | 0.55±0.006ab | 0.55±0.004a | 0.53±0.002ab | 0.53±0.006a |  |
| 3 | 0.53±0.021ab | 0.55±0.007ab | 0.53±0.002ab | 0.53±0.003ab | 0.54±0.001ab | 0.55±0.002a | 0.54±0.007ab | 0.52±0.004ab | 0.53±0.002a |  |
| 4 | 0.54±0.006ab | 0.54±0.002cd | 0.54±0.020a | 0.53±0.004ab | 0.54±0.005ab | 0.55±0.012a | 0.53±0.010bc | 0.53±0.009ab | 0.54±0.008a |  |
| 5 | 0.56±0.013a | 0.55±0.002ab | 0.52±0.02ab | 0.53±0.014ab | 0.53±0.006bc | 0.55±0.003a | 0.54±0.007ab | 0.53±0.002ab | 0.55±0.019a |  |
| 6 | 0.54±0.004ab | 0.54±0.003bc | 0.53±0.005ab | 0.53±0.003ab | 0.53±0.002ab | 0.54±0.005bc | 0.52±0.008c | 0.52±0.004b | 0.54±0.016a |  |
| 7 | 0.54±0.003ab | 0.53±0.003de | 0.52±0.08ab | 0.53±0.004ab | 0.53±0.010bc | 0.53±0.007cd | 0.48±0.005d | 0.52±0.001ab | 0.49±0.006b |  |
| 8 | 0.53±0.034abc | 0.53±0.006e | 0.51±0.002ab | 0.52±0.005ab | 0.51±0.004de | 0.53±0.009cd | 0.44±0.007e | 0.49±0.002c | 0.49±0.004b |  |
| 9 | 0.52±0.006bc | 0.52±0.006f | 0.53±0.025ab | 0.50±0.005cd | 0.50±0.007e | 0.52±0.005de | 0.45±0.009e | 0.49±0.003d | 0.45±0.005bc |  |
| 10 | 0.51±0.020c | 0.52±0.007f | 0.51±0.002ab | 0.47±0.004e | 0.46±0.004f | 0.52±0.006e | 0.42±0.025f | 0.46±0.003e | 0.44±0.007c |  |
| 11 | 0.48±0.005d | 0.50±0.004g | 0.49±0.011b | 0.46±0.002e | 0.47±0.006f | 0.49±0.0045f | 0.40±0.013g | 0.45±0.008f | 0.42±0.020d |  |
| 12 | 0.43±0.005e | 0.491±0.004h | 0.45±0.005c | 0.44±0.001f | 0.46±0.004f | 0.48±0.010f | 0.40±0.005g | 0.43±0.005g | 0.40±0.008e |  |
| 13 | 0.37±0.006f | 0.47±0.008i | 0.42±0.006c | 0.42±0.007fg | 0.42±0.006g | 0.47±0.006g | 0.37±0.008h | 0.41±0.003h | 0.37±0.007f |  |
| 14 | 0.34±0.002g | 0.44±0.002j | 0.20±0.009d | 0.399±0.004g | 0.39±0.001h | 0.43±0.0004h | 0.36±0.007h | 0.37±0.008i | 0.34±0.005f |  |

Leaching of polyphenols of Zhengyan (A1, A2, A3), Banyan (B1, B2, B3) and Zhouyan (C1, C2, C3) tea samples. BT represents steeping times (cuppages); S represents Samples. Data are presented as means ± SD of three replicates. Different letters indicate significant differences at P < 0.05.

| **Table S5.** Leaching of amino acids (μg /mL) | | | | | | | | | |
| --- | --- | --- | --- | --- | --- | --- | --- | --- | --- |
| BT/S | A1 | A2 | A3 | B1 | B2 | B3 | C1 | C2 | C3 |
| 1 | 0.35±0.023a | 0.4±0.017a | 0.25±0.000b | 0.34±0.003a | 0.27±0.005a | 0.45±0.005a | 0.29±0.009ab | 0.29±0.005a | 0.33±0.007a |
| 2 | 0.36±0.004a | 0.38±0.000b | 0.29±0.002a | 0.35±0.002a | 0.26±0.007a | 0.45±0.002a | 0.34±0.002a | 0.28±0.003b | 0.35±0.035a |
| 3 | 0.27±0.006bc | 0.35±0.012c | 0.25±0.005bc | 0.30±0.007ab | 0.25±0.002b | 0.48±0.018a | 0.26±0.014abc | 0.24±0.002c | 0.32±0.012a |
| 4 | 0.28±0.004b | 0.27±0.000d | 0.22±0.002c | 0.26±0.000abc | 0.24±0.003b | 0.44±0.019a | 0.23±0.000bc | 0.25±0.0035c | 0.26±0.002b |
| 5 | 0.23±0.257bcd | 0.26±0.012d | 0.24±0.001d | 0.27±0.013abc | 0.22±0.003c | 0.34±0.005b | 0.25±0.000abc | 0.25±0.0045c | 0.24±0.002b |
| 6 | 0.23±0.003bcd | 0.23±0.000e | 0.21±0.003d | 0.23±0.00bc | 0.21±0.000d | 0.28±0.011c | 0.24±0.013bc | 0.22±0.0017d | 0.23±0.013b |
| 7 | 0.22±0.004cd | 0.21±0.017f | 0.20±0.006e | 0.21±0.003c | 0.20±0.003d | 0.24±0.007cd | 0.20±0.003bcd | 0.22±0.0047d | 0.22±0.012b |
| 8 | 0.24±0.003bcd | 0.2±0.000fg | 0.20±0.006e | 0.20±0.004c | 0.20±0.012de | 0.23±0.006cd | 0.20±0.001cd | 0.21±0.013e | 0.20±0.003b |
| 9 | 0.20±0.005d | 0.2±0.000fg | 0.19±0.000f | 0.20±0.004c | 0.20±0.013d | 0.22±0.006cd | 0.19±0.000cd | 0.20±0.004ef | 0.22±0.006b |
| 10 | 0.20±0.002d | 0.2±0.000fg | 0.19±0.014g | 0.19±0.006c | 0.19±0.007ef | 0.22±0.006cd | 0.12±0.104d | 0.19±0.003fg | 0.2±0.000b |
| 11 | 0.19±0.005d | 0.2±0.000fg | 0.18±0.000g | 0.07±0.119d | 0.18±0.000f | 0.21±0.006d | 0.12±0.109d | 0.19±0.004g | 0.2±0.002b |
| 12 | 0.06±0.111e | 0.19±0.006fgh | 0.00±0.000h | 0.08±0.133d | 0.00±0.000g | 0.07±0.115f | 0.12±0.106d | 0.00±0.000h | 0.13±0.011b |
| 13 | 0.00±0.000f | 0.19±0.012gh | 0.00±0.000h | 0.00±0.000d | 0.00±0.000g | 0.00±0.000f | 0.00±0.000e | 0.00±0.000h | 0.00±0.000c |
| 14 | 0.00±0.000f | 0.18±0.006h | 0.00±0.000h | 0.00±0.000d | 0.00±0.000g | 0.00±0.000f | 0.00±0.000e | 0.00±0.000h | 0.00±0.000c |

Leaching of amino acids of Zhengyan (A1, A2, A3), Banyan (B1, B2, B3) and Zhouyan (C1, C2, C3) tea samples. BT represents steeping times (cuppages); S represents Samples. Data are presented as means ± SD of three replicates. Different letters indicate significant differences at P < 0.05.

| **Table S6.** Leaching of caffeine (μg /mL) | | | | | | | | | |
| --- | --- | --- | --- | --- | --- | --- | --- | --- | --- |
| BT/s | A1 | A2 | A3 | B1 | B2 | B3 | C1 | C2 | C3 |
| 1 | 312±10.58b | 333.33±6.11c | 147.33±12.01d | 304±5.29c | 320.67±10.07b | 244.00±13.11c | 308.33±7.64b | 280.33±12.50c | 326.67±26.03b |
| 2 | 366.33±9.5a | 467.67±11.06a | 329±17.00a | 454.33±4.16a | 437.00±7.55a | 387.00±12.29a | 418.00±2.65a | 426.33±8.51a | 431.00±11.53a |
| 3 | 274±6.56c | 347.00±8.19b | 292.33±11.01b | 360±4.00b | 329.67±7.57b | 299.33±20.84b | 284.00±3.61c | 313.33±7.64b | 324.33±22.72b |
| 4 | 162.67±4.73d | 218.33±16.26d | 208.33±8.02c | 225.33±4.51d | 214.67±13.05c | 214.67±16.17d | 155.67±4.93d | 217.67±10.02d | 221.00±17.06c |
| 5 | 108.67±7.77e | 129.67±4.51e | 129.67±7.77e | 155.67±5.13e | 129.00±8.54d | 154.67±9.50e | 105.67±6.03e | 145.33±10.50e | 127.00±7.55d |
| 6 | 65.33±5.51f | 105.67±6.03f | 77.33±7.51f | 84±3.61f | 78.33.8.02e | 119.00±15.72f | 52.67±3.06f | 85.33±5.51f | 93.33±14.30e |
| 7 | 38±3.61g | 65.00±6.25g | 43.67±8.50g | 54.67±4.51g | 48.33±8.51f | 89.33±11.50g | 31.67±2.08g | 49.00±9.00g | 65.00±9.54f |
| 8 | 21.67±1.16h | 44.67±4.51h | 28.33±8.51h | 31.67±2.08h | 31.00±7.56f | 54.67±9.51h | 16.33±3.79h | 27.67±7.64h | 38.00±3.46g |
| 9 | 10.33±2.52i | 29.67±6.03i | 17.67±7.64hi | 22.67±4.04i | 20.67±4.73g | 37.00±4.58hi | 9.33±1.53i | 18.00±5.57hi | 18.67±4.51gh |
| 10 | 5.67±0.58ij | 14.33±3.79j | 11.00±4.58ij | 13.67±3.21j | 7.00±2.00h | 21.33±6.03ij | 4.33±0.58ij | 10.67±3.79ij | 13.67±4.04i |
| 11 | 4.67±0.58ij | 9.33±3.06jk | 6.33±2.52ij | 5.00±1.00k | 3.67±2.09h | 16.67±7.02ij | 3.33±1.53ij | 5.00±2.00j | 8.00±3.61i |
| 12 | 1.37±0.15ij | 6.00±1.00jk | 5.33±3.51ij | 5.33±1.53k | 2.53±1.50h | 11.00±2.65ij | 1.23±0.25j | 2.67±2.08j | 6.00±2.65i |
| 13 | 0.9±0.10j | 3±1.00jk | 3.00±1.00ij | 3.00±1.00k | 1.20±0.63h | 6.00±2.00k | 0.30±0.10j | 1.00±0.40j | 2.13±0.42i |
| 14 | 0.37±0.15j | 1.50±0.50jk | 1.67±0.58j | 2.33±0.58k | 0.87±0.35h | 2.33±1.53k | 0.20±0.10j | 0.30±0.27j | 0.56±0.41i |

Leaching of caffeine of Zhengyan (A1, A2, A3), Banyan (B1, B2, B3) and Zhouyan (C1, C2, C3) tea samples. BT represents steeping times (cuppages); S represents samples. Data are presented as means ± SD of three replicates. Different letters indicate significant differences at P < 0.05.
