# Supplementary material for: Prediction of suitable brewing cuppages of Dahongpao tea based on chemical composition, liquor colour and sensory quality in different brewing
Source: Sci Rep. 2020 Jan 22;10:945. doi: 10.1038/s41598-020-57623-5 (PMC6976566; doi:10.1038/s41598-020-57623-5)
Supplement: Supplementary file 2 — Supplementary Information Doc.-Figure. [file 41598_2020_57623_MOESM2_ESM.docx]

**Prediction of suitable brewing cuppages of Dahongpao tea based on chemical composition, liquor colour and sensory quality in different brewing**

Sifeng Zhang, Yiqing Yang, Xiaofang Cheng, Kuberan Thangaraj, Emmanuel Arkorful, Xuan Chen, Xinghui Li*

Institute of Tea Science, Nanjing Agricultural University, Weigang No.1, 210095, Nanjing, China

* Corresponding author: Xinghui Li

Email: lxh@njau.edu.cn

Tel/fax: +86 25 8439 6651

Sifeng Zhang 2016104090@njau.edu.cn

Yiqing Yang 14216111@njau.edu.cn

Xiaofang Cheng 14217131@njau.edu.cn

Kuberan Thangaraj tku2010@gmail.com

Emmanuel Arkorful emmamidnite@hotmail.com

Xuan Chen chenxuan@njau.edu.cn

Xinghui Li lxh@njau.edu.cn

**
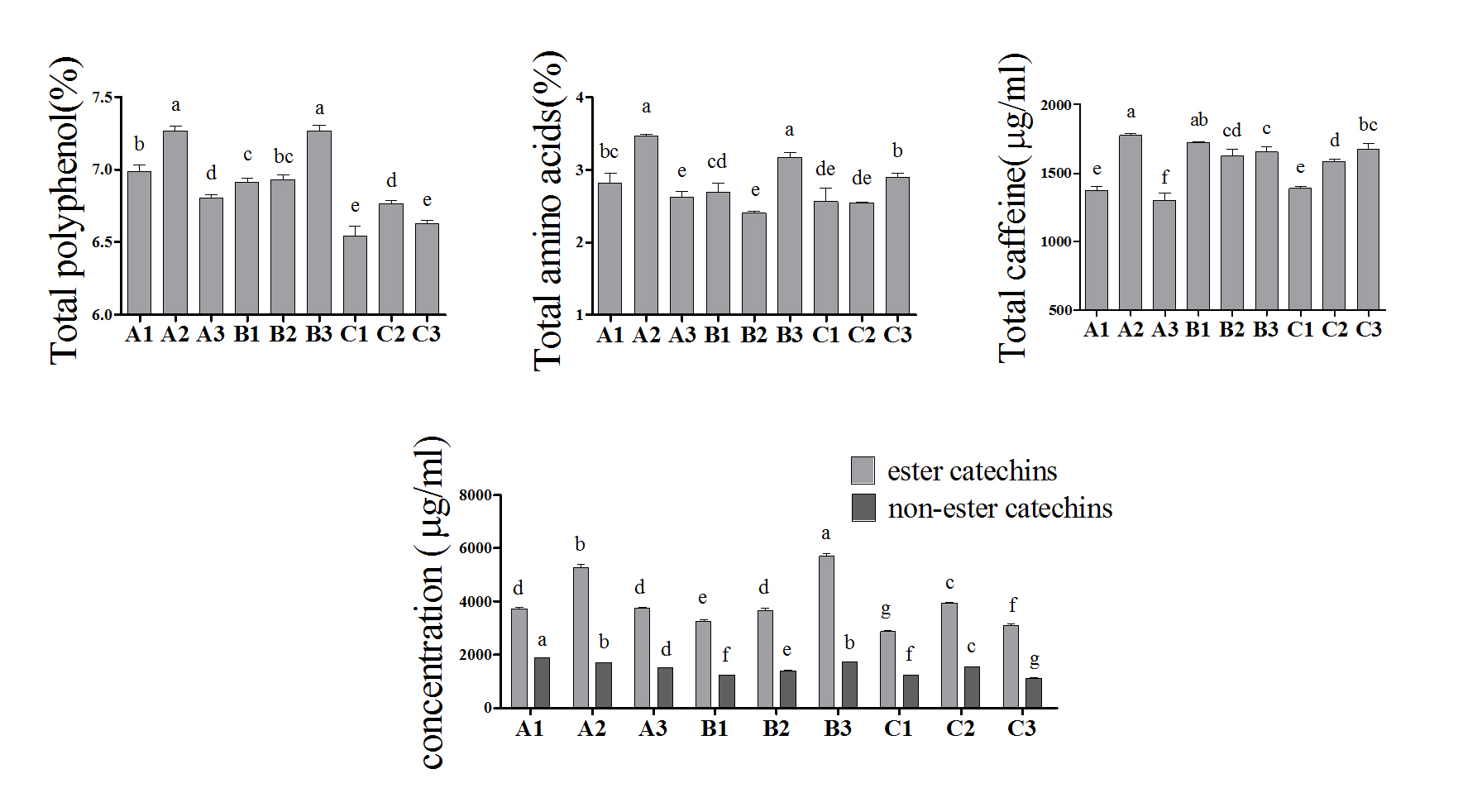
**

**Figure S1.** Total polyphenol, amino acid, caffeine, ester and non-ester catechins contents of Zhengyan (A1, A2, A3), Banyan (B1, B2, B3), and Zhouyan (C1, C2, C3) tea samples. Data are presented as means ± SD. Different letters indicate significant differences at P < 0.05.

**
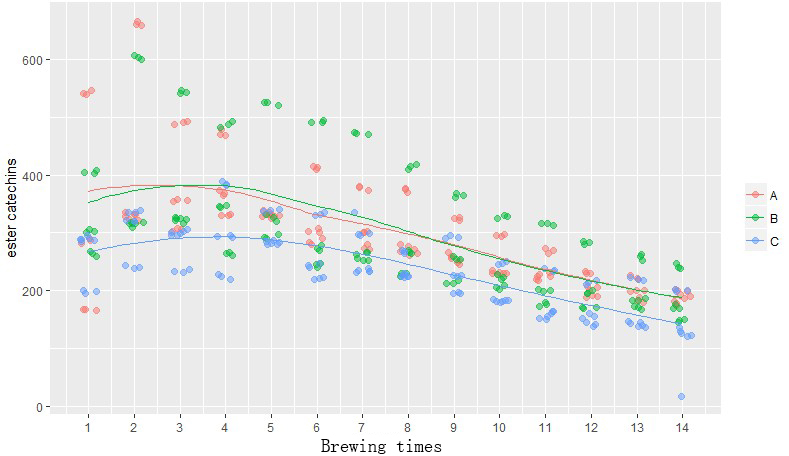
**

**Figure S2.** Principal component analysis of ester catechins in Zhengyan (A1, A2, A3), Banyan (B1, B2, B3), and Zhouyan (C1, C2, C3) tea samples.

**
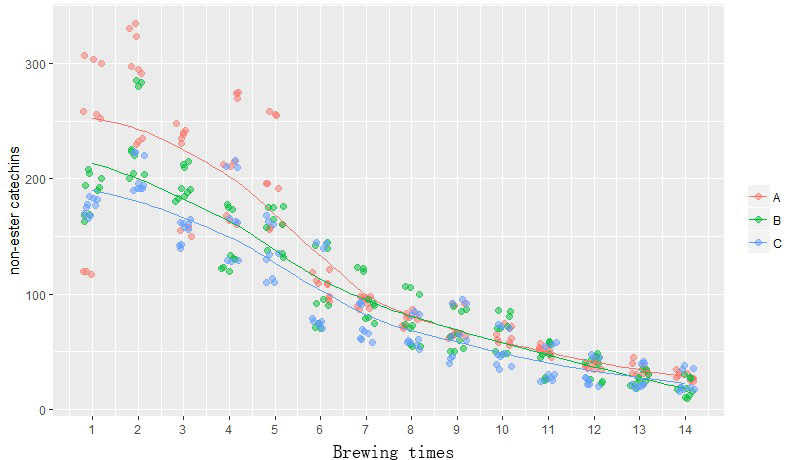
**

**Figure S3.** Principal component analysis of non-ester catechins in Zhengyan (A1, A2, A3), Banyan (B1, B2, B3), and Zhouyan (C1, C2, C3) tea samples.

**
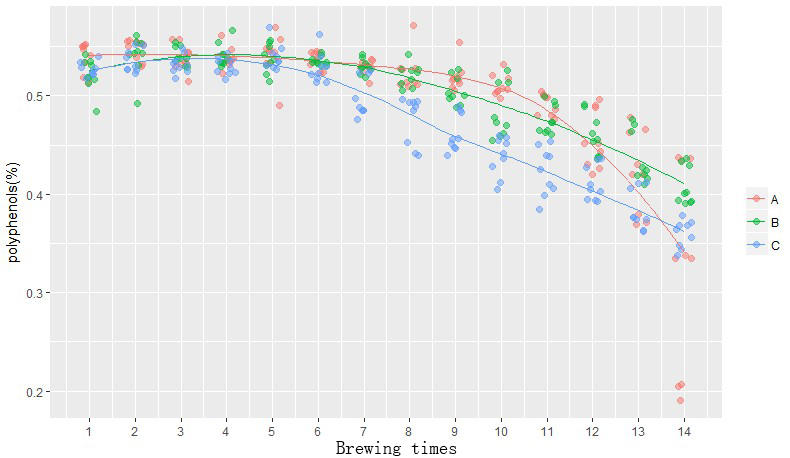
**

**Figure S4.** Principal component analysis of total polyphenols in Zhengyan (A1, A2, A3), Banyan (B1, B2, B3) and Zhouyan (C1, C2, C3) tea samples.

**
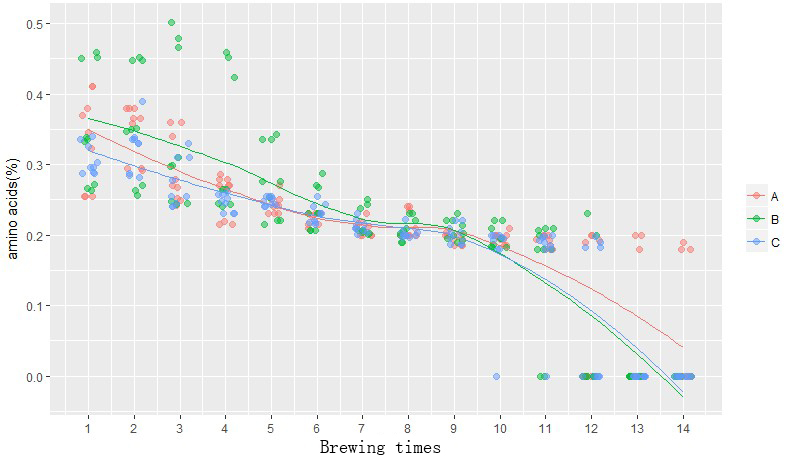
**

**Figure S5.** Principal component analysis of amino acids levels in Zhengyan (A1, A2, A3), Banyan (B1, B2, B3) and Zhouyan (C1, C2, C3) tea samples.

**
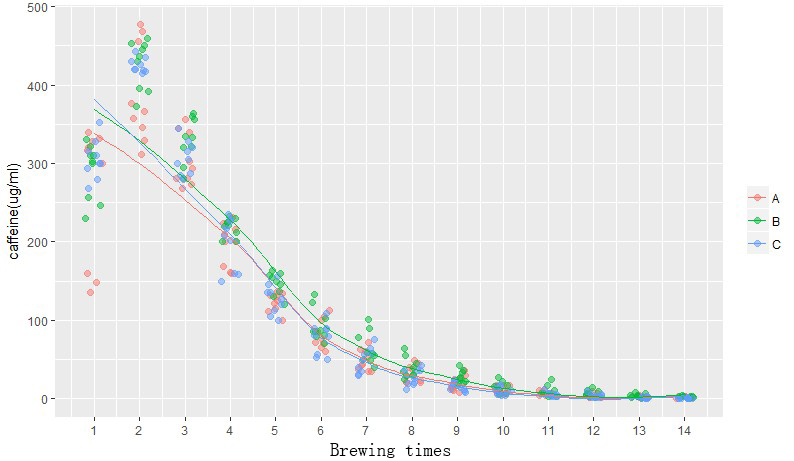
**

**Figure S6.** Principal component analysis of caffeine content in Zhengyan (A1, A2, A3), Banyan (B1, B2, B3) and Zhouyan (C1, C2, C3) tea samples.

**
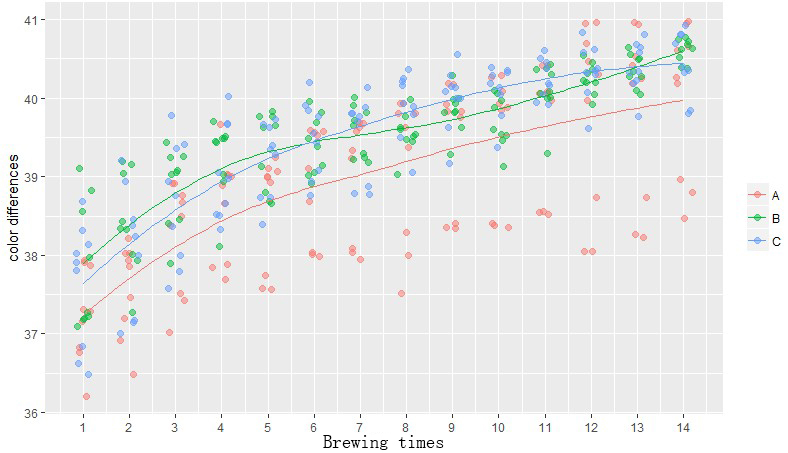
**

**Figure S7.** Principal component analysis of colour changes of Zhengyan (A1, A2, A3), Banyan (B1, B2, B3) and Zhouyan (C1, C2, C3) tea samples.

**
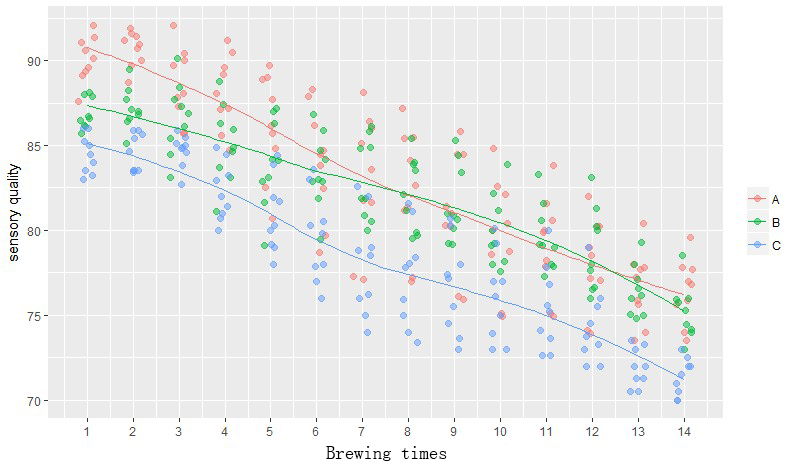
**

**Figure S8.** Principal component analysis of sensory quality of Zhengyan (A1, A2, A3), Banyan (B1, B2, B3) and Zhouyan (C1, C2, C3) tea samples.
